# Rapid cycle deliberate practice *versus* postsimulation debriefing in pediatric cardiopulmonary resuscitation training: a randomized controlled study

**DOI:** 10.31744/einstein_journal/2024AO0825

**Published:** 2024-07-17

**Authors:** Laila Pinto Coelho, Sylvia Costa Lima Farhat, Rafael da Silva Giannasi Severini, Ana Carolina Amarante Souza, Katharina Reichmann Rodrigues, Fernanda Paixão Silveira Bello, Claudio Schvartsman, Thomaz Bittencourt Couto

**Affiliations:** 1 Faculdade de Medicina Universidade de São Paulo São Paulo SP Brazil Faculdade de Medicina, Universidade de São Paulo, São Paulo, SP, Brazil.; 2 Hospital das Clínicas Faculdade de Medicina Universidade de São Paulo São Paulo SP Brazil Instituto da Criança (ICr), Hospital das Clínicas, Faculdade de Medicina, Universidade de São Paulo, São Paulo, SP, Brazil.; 3 Hospital Israelita Albert Einstein São Paulo SP Brazil Hospital Israelita Albert Einstein, São Paulo, SP, Brazil.; 4 Faculdade Israelita de Ciências da Saúde Albert Einstein Hospital Israelita Albert Einstein São Paulo SP Brazil Faculdade Israelita de Ciências da Saúde Albert Einstein, Hospital Israelita Albert Einstein, São Paulo, SP, Brazil.

**Keywords:** Patient simulation, Simulation training, Cardiopulmonary resuscitation, Pediatric emergency medicine, Internship and residency

## Abstract

**Objective:**

Simulation plays an important role in cardiopulmonary resuscitation training. Comparing postsimulation debriefing with rapid cycle deliberate practice could help determine the best simulation strategy for pediatric cardiopulmonary resuscitation training among pediatric residents.

**Methods:**

This is a single-blind, prospective, randomized controlled study. First- and second year pediatric residents were enrolled and randomized into two groups (1:1 ratio): rapid cycle deliberate practice group (intervention) or postsimulation debriefing group (control). They participated in two rounds of simulated pediatric cardiopulmonary arrest to assess the simulated pediatric cardiopulmonary resuscitation performance gain (round 1) and retention after a 5-6 week washout period (round 2). Scenarios were video-recorded and analyzed by blinded evaluators. The main outcome was the time to initiation of chest compressions. Secondary outcomes included time to recognize a cardiopulmonary arrest, time to recognize a shockable rhythm, time to defibrillation, time to initiation of chest compressions after defibrillation, and chest compression fraction.

**Results:**

Sixteen groups participated in the first round and fifteen groups in the second one. Time to intiation of chest compressions decreased from preintervention scenario to the round 1 testing scenario and increased from round 1 to round 2 testing scenario. However, no interaction effects nor group effects were observed (p=0.885 and p=0.329, respectively). There were no significant differences between the two groups regarding the secondary outcomes.

**Conclusion:**

Despite an overall improvement in simulated pediatric cardiopulmonary resuscitation performance, we did not observe significant differences between the two groups regarding the analyzed variables. The decline in simulated pediatric cardiopulmonary resuscitation performance after 5 weeks suggests the need for shorter time intervals between training sessions.

## In Brief

This single-blind, prospective, randomized controlled study compared postsimulation debriefing and rapid cycle deliberate practice to determine the optimal cardiopulmonary resuscitation training method for pediatric residents. No significant differences were observed between the two simulation strategies regarding time to the performance of critical tasks and skill retention in simulated pediatric cardiopulmonary resuscitation.

## Highlights

Improvement in the performance of simulated pediatric cardiopulmonary resuscitation was observed after postsimulation debriefing and rapid cycle deliberate practice sessions.

Postsimulation debriefing and rapid cycle deliberate practice are suitable for cardiopulmonary resuscitation training among pediatric residents.

Retention of knowledge and skills deteriorated 5 week safter training.

## Graphical abstract

**Figure:**
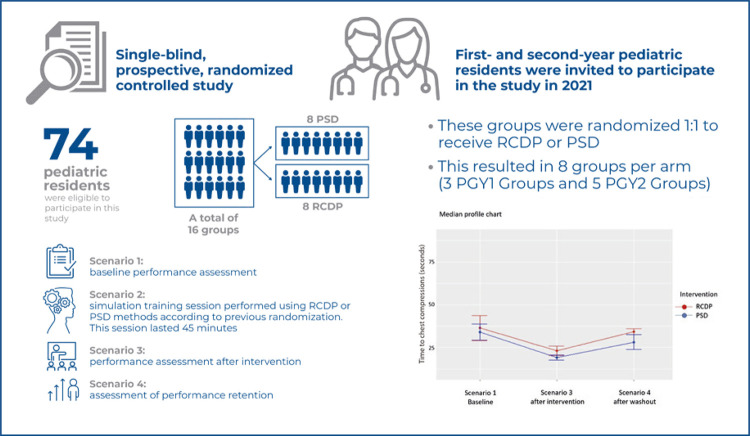

## INTRODUCTION

Pediatric cardiopulmonary arrest (CPA) is a rare event with a poor prognosis.^(1-3)^ Several studies have demonstrated the importance of high-quality cardiopulmonary resuscitation (CPR) for pediatric CPA outcomes.^(3-5)^ However, despite well-established algorithms for pediatric CPA management and consolidated training courses, healthcare providers, including pediatric residents, do not always perform adequately when confronted with pediatric CPA.^(6-8)^ This highlights the importance of determining the best educational strategy and frequency for pediatric CPR training.

Simulation-based medical education has been shown to be a valuable tool to improve knowledge, procedural skills, and behaviors necessary for attaining a high performance in low-frequency, high-acuity events; hence playing an important role in pediatric CPR training.^(9-11)^ Traditional scenario-based simulation with postsimulation debriefing (PSD) has been widely used for pediatric CPR training.^(9)^ In PSD, a case scenario is presented learners engage in a simulation case, and a debriefing session follows. The debriefing session allows reflection and discussion of what went well and what could be improved regarding the scenario, encouraging reflective practice and experiential learning in a safe environment.

However, once the debriefing session ends, there is no more time to practice what has just been discussed. In addition, the time allocated to debrief is usually longer than the time allocated for the scenarios, resulting in fewer opportunities to practice. Skill and knowledge decay over time have also been observed after PSD training.^(12)^ These could be seen as a limitation of this method in regards to CPR training, considering the importance of repetitive practice to master critical tasks, procedural skills, adherence to protocols and team choreography.

Rapid cycle deliberate practice (RCDP) is a simulation model, introduced by Hunt et al. that combines repetitive practice, targeted feedback, mastery learning, and progressive difficulty.^(13)^ It is based on three main principles. First it involves providingmultiple practice opportunities to create muscle memory through overlearning and automatization. Second it entails offeringdirect feedback from experts. Third, it involves establishing a psychologically safe environment where participants can welcome this feedback.^(13-15)^

During RCDP, the simulation session consists of several consecutive rounds with increasing degrees of difficulty. To pass from one round to the next, specific, predefined learning objectives must be achieved and mastered. Each round is simulated several times to enable repetitive practice until specific skills and performance are mastered. During each round, after the case scenario is presented, the learners engage in simulation. Whenever a performance gap or an opportunity for improvement is identified by the facilitator, the scenario is paused, direct feedback is provided, and the scenario is repeated to allow the participants to try again and do it “the right waythu” (s creating a feedback-replay loop).^(15)^ The goal is to provide participants with the opportunity to practice until they have attained mastery. When mastery is achieved, another round with subsequent learning objectives and increased difficulty is begun. This strategy differs from PSD, which emphasizes reflection and discussion after scenario completion.

Rapid cycle deliberate practice is associated with improvement in neonatology and pediatric resuscitation skills, critical performance, and teamwork.^(16-20)^ Furthermore, compared with PSD, RCDP could potentially improve the retention of pediatric resuscitation skills.^(20)^

Both simulation methods have been associated with positive outcomes in simulated pediatric CPR performance immediately after training. However, RCDP has been shown to be superior to PSD for pediatric CPR training in terms of specific pediatric resuscitation outcomes, such as improvement in time to defibrillation and team performance (human factors).^(18,20)^ Nevertheless, a declinein knowledge and performance has also been demonstrated following training. This is clinically relevant because of the impact of high-quality CPR on pediatric CPA outcomes.^(16,17,21,22)^ There is limited data on the retention of simulated pediatric CPR performance when comparing both methods.

We hypothesized that rapid cycle deliberate practice strategy would be superior to postsimulation debriefing in this regard.

## OBJECTIVE

Our goal was to evaluate both simulation approaches for pediatric cardiopulmonary resuscitation training among pediatric residents and to identify the most effective simulation strategy to enhance and sustain simulated pediatric cardiopulmonary resuscitation performance.

## METHODS

This is a single-blind, prospective, randomized controlled study.

### Setting

Our pediatric residency program is based at a large, urban, Tertiary Care University Hospital pediatric affiliated Hospital *(Instituto da Criança, Hospital das Clínicas, Faculdade de Medicina, Universidade de São Paulo* - ICR-HCFMUSP) in São Paulo, Brazil.

This study was conducted at the Simulation Center of a Tertiary Care University Hospital in São Paulo, Brazil, (HC-FMUSP) and in situ in the emergency room of the pediatric inpatient unit of the Children’s Institute of ICR-HCFMUSP.

### Study population

First- and second-year pediatric residents at the *Hospital das Clínicas da Faculdade de Medicina da Universidade de São Paulo* were invited to participate in this study in 2021. A convenience sample of 80 residents was expected (40 first-year residents and 40 second-year residents). All residents who agreed to participate, only entered the study once. The schedules of first- and second-year pediatric residents comprised two emergency department rotations per year that were separated by a 5-6-week interval. Residents rotated in groups of four to five and remained in the same group throughout the year. All residents participated in this study during their pediatric emergency department rotation.

### Institutional board review approval

The institutional review board approved this study, and written informed consent was obtained from all participants (Certificate of Presentation for Ethical Consideration from the *Hospital das Clínicas* of the *Faculdade de Medicina* of the *Universidade de São Paulo*; CAAE: 39402520.2.0000.0068; #4.583.449).

### Study design

**Figure 1:**
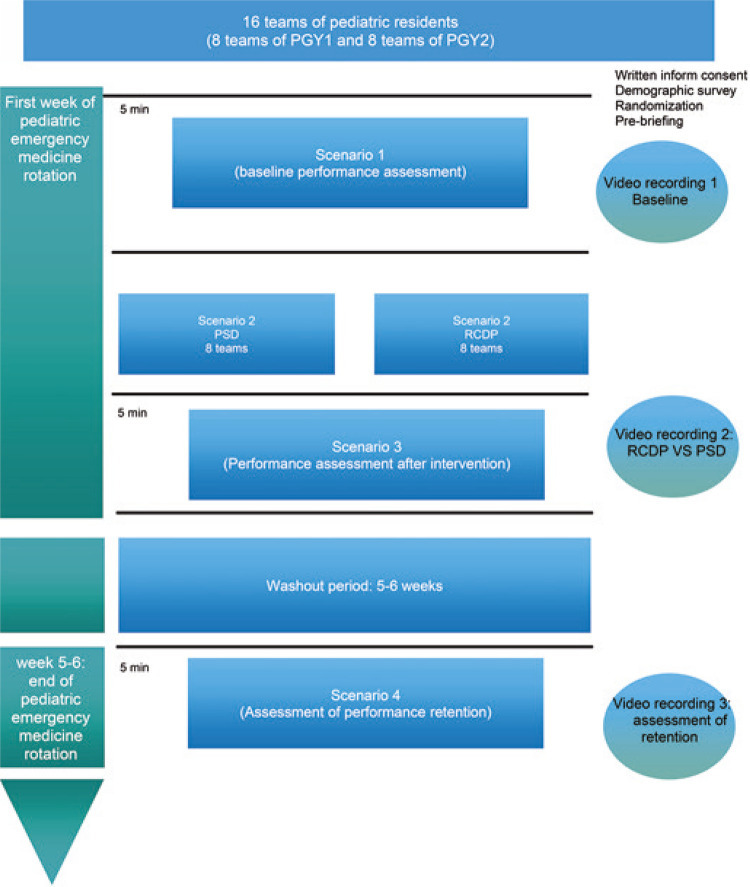
The teams and simulations are described

PSD: postsimulation debriefing; RCDP: rapid cycle deliberate practice; PGY1: postgraduate year 1 (first-year pediatric residents); PGY2: postgraduate year 2 (second-year pediatric residents).

First- and second-year pediatric residents were enrolled and randomized (1:1 ratio) to either the RCDP arm (intervention group) or the PSD arm (control group). Groups of four to six residents were formed in each arm and these groups remained the same throughout the study period. After randomization, the participants completed a demographic questionnaire. Then, they participated in two rounds of simulated pediatric CPA so that the simulated pediatric CPR performance improvement (round 1) and knowledge and skills retention after a 5-6-week washout period (round 2) could be assessed. Both rounds were preceded by a pre-briefing session to review the learning objectives, provide orientation of the simulation environment and mannequin,establish a psychologically safe environment and a confidentiality contract (Figure 1).

Round 1 consisted of three consecutive simulated pediatric CPA scenarios. Scenario 1 lasted 5 minutes, proceeded without any instructor interference, and comprised a simulated pediatric CPA (shockable rhythm). The goal was to assess the baseline pediatric CPR performance. Scenario 2 lasted 45 minutes, and comprised simulation training sessions using RCDP or PSD according to randomization. Scenario 3 lasted 5 min and proceeded without any instructor interference. The goal was to assess the simulated pediatric CPR performance immediately after the training session.

Round 2 was conducted after a washout period of 5-6 weeks and consisted of a single scenario of simulated pediatric CPA (shockable rhythm) that occurred without any instructor interference. The goal was to assess the retention of simulated pediatric CPR skills.

During these two rounds, the participants (four to six participants per group) rotated between the following roles: team leader, compressor, airway manager, monitor/defibrillator, intravenous (IV)/intraosseous (IO) medication provider, and time recorder. The instructor assisted the residents with IV/IO medication and time recording when necessary. These roles were evenly distributed among the residents and rotated during each scenario.

Learning objectives were based on the American Heart Association’s recommendations and algorithms for pediatric CPR. A total of two instructors were involved in all simulations, and each scenario was facilitated by one instructor. These instructors were experienced simulation facilitators who worked in the simulation center and pediatric emergency department. They were trained in PSD and RCDP methods, and they had experience providing PSD and RCDP training to healthcare providers.

The simulations were conducted at the simulation center or in situ in the emergency room of the pediatric inpatient unit. Both were fully equipped with an emergency cart and Philips HeartStart XL defibrillator (Eindhoven, Netherlands). The resusci baby Laerdal, with a SimPad SkillReporter (Laerdal) were used in all simulation sessions. Scenarios 1, 3, and 4 were video-recorded and further analyzed by blinded evaluators.

### Simulation curriculum design for scenario 2 (training session)

#### Simulation with PSD

Scenario 2 comprised a pediatric patient who arrived at the pediatric emergency department in respiratory arrest, progressed to CPA with nonshockable rhythm and subsequently to CPA with shockable rhythm. The scenario lasted 15 minutes and was followed by a debriefing session of approximately 30 minutes. The debriefing was pre-scripted to standardize the discussion of specific learning objectives across groups. PEARLS (Promoting Excellence and Reflective Learning in Simulation) framework was used.^(23)^

#### Simulation with RCDP

During a 45-min period, the residents participated in three consecutive scenarios with increasing levels of difficulty. First, they were exposed to a simulated pediatric patient who had arrived at the pediatric emergency department with respiratory arrest, followed by a simulated pediatric patient with CPA with nonshockable rhythm and a simulated pediatric patient with CPA with shockable rhythm. Specific learning objectives had to be mastered before progressing from one scenario to the next. Whenever the instructor identified a performance gap, the scenario was paused and targeted feedback was provided. Then, the scenario was resumed from the point at which it was paused or slightly before that point to provide the participants with the opportunity to try again until mastery was achieved. To standardize the RCDP sessions and maintain similar learning objectives across groups, a feedback sheet with predefined “hard and soft stops” was used for guidance.

## Outcomes

The main outcome was the time to initiation of chest compressions. Secondary outcomes included time to recognition of CPA, time to recognition of the rhythm on the monitor, time to defibrillation, time to initiation of chest compressions after defibrillation, and the chest compression fraction. The quality of the chest compressions was also assessed.

## Data collection

Video recordings of scenarios 1, 3, and 4 were further analyzed by four evaluators who were blinded to the randomization arm, as well as to the scenario’s number and timing (pre/post intervention). One of these evaluators was also an instructor during the scenarios; however, he did not analyze the videos of the simulation sessions that he had supervised. Each evaluator analyzed an average of 12 video recordings by completing a pre-established checklist that encompassed our outcome measures. The chest compression rate, chest compression depth, chest recoil, and chest compression fractions were extracted from the SimPad SkillReporter.

## Statistical analysis

During the descriptive analysis, relative and absolute frequencies were used for qualitative variables. The means, medians, standard deviations, percentiles, and maximum and minimum values were used for quantitative variables.

Comparisons of the RCDP and PSD Groups were performed using Fisher’s exact test or the χ^2^ test for qualitative variables. For quantitative variables, either Student’s *t*-test or the nonparametric Mann-Whitney U test was performed, depending on the distribution. The distribution was assessed using descriptive statistics, measures of skewness and kurtosis, normal probability plots, and the Shapiro-Wilk test for normality. The progress of the simulated pediatric CPR performance between groups from scenario 1 (baseline) to scenario 3 (immediately after the intervention) and scenario 4 (after the washout period) was analyzed using repeated measures analysis of variance (ANOVA) models. These models were used to investigate between-group comparisons and within-group comparisons (across scenarios). Analyses were conducted using Jamovi software version 2.3.18 and R version 4.2.2. A standard level of statistical significance of 5% was adopted for all tests.

## RESULTS

### Participants

Seventy-four pediatric residents were eligible to participate in this study. After they were enrolled, they were allocated to groups of three to six participants according to their year of residency and pediatric emergency medicine rotation schedule. This resulted in 16 groups (6 groups of first-year pediatric residents and 10 groups of second-year pediatric residents). These groups were randomized using a 1:1 ratio to receive either RCDP training or PSD training. This resulted in eight groups per arm (three groups of first-year pediatric residents and five groups of second-year pediatric residents).

**Figure 2:**
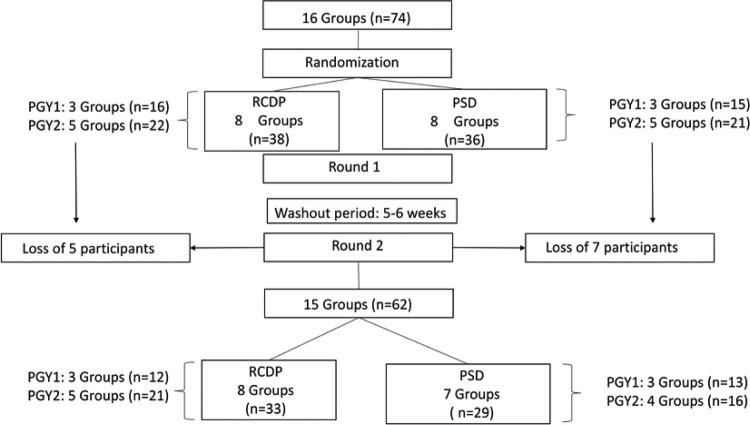
Flow of the study participants

PSD: postsimulation debriefing; RCDP: rapid cycle deliberate practice; PGY1: postgraduate year 1 (first-year pediatric residents); PGY2: postgraduate year 2 (second-year pediatric residents).

After a washout period of 5-6 weeks, 15 groups of residents (6 groups of first-year pediatric residents and 9 groups of second-year pediatric residents) participated in round 2. As the residents participated in our study during their pediatric emergency medicine rotation, some of them could not participate in round 2 because they had to attend to real emergencies. Therefore, 12 participants could not be evaluated during this phase of our study (round 2), and one group of second-year pediatric residents who were initially allocated to the PSD Group was lost to follow-up. Ultimately, we analyzed the data of 15 groups (n=62 pediatric residents) (Figure 2).

The participants’ characteristics are presented in table 1. Sixty-five participants completed the demographic questionnaire. There were no significant differences between the baseline characteristics (such as previous simulation training experience, overall clinical experience, and pediatric advanced life support certification).

**Table 1:** Participants’ demographics

Variables	Total number of answers (n=65)	Groups			p value

		PSD (n=32)		RCDP (n=33)	
Female participants, n (%)	54 (83.1)	28 (87.5)		26 (78.7)	0.349^†^
Median age, years, (standard deviation)	25.6 (1.61)	25.57 (1.47)		25.71 (1.75)	0.732^‡^
Year of graduation, n (%)					
2017	3 (4.6)	3 (9.37)		-	
2018	7 (10.8)	4 (12.5)		3 (9.1)	0.456^§^
2019	26 (40)	13 (40.6)		13 (39.4)	
2020	23 (35.4)	10 (31.3)		13 (39.4)	
2021	6 (9.2)	2 (6.2)		4 (12.1)	
Previous training (%)					
BLS	21 (32.3)	12 (37.5)		9 (27.3)	0.378^†^
PALS	35 (53.8)	18 (56.2)		17 (51.5)	0.702^†^
ACLS	52 (80)	27 (84.3)		25 (75.8)	0.385^†^
Previous experience with simulation, n (%)	63 (96.9)	31 (96.8)		32 (96.9)	>0.999^§^
Participation in real pediatric CPA	23 (35.4)	10 (31.3)		13 (39.4)	0.492^†^
Participation in simulated pediatric CPA scenarios	59 (90.8)	29 (90.6)		30 (90.9)	0.999^†^
Already performed defibrillation for a real patient	12 (18.5)	6 (18.8)		6 (18.2)	0.953^†^
Using a scale from 1 (not confident at all) to 5* (absolutely confident), how confident are you in managing a pediatric CPA? n (%)^≠^					
1 (Not confident at all)	12 (18.5)	4 (12.5)	8 (24.2)	0.170^†^	
2 (Somewhat confident)	38 (58.5)	17 (531)	21 (63.6)		
3 (Confident)	13 (23)	9 (34.4)	4 (12.2)		

^†^ χ^2^ test; ^‡^ Unpaired Student’s *t* test; ^§^ Fisher’s exact test; * No participants graded their confidence as 4 or 5 using this scale; ^≠^ Two participants did not answer this question.

ACLS: advanced cardiovascular life support; BLS: basic life support; CPA: cardiopulmonary arrest; PALS: pediatric advanced life support; PSD: postsimulation debriefing; RCDP: rapid cycle deliberate practice.

### Performance

#### Primary outcome

**Figure 3:**
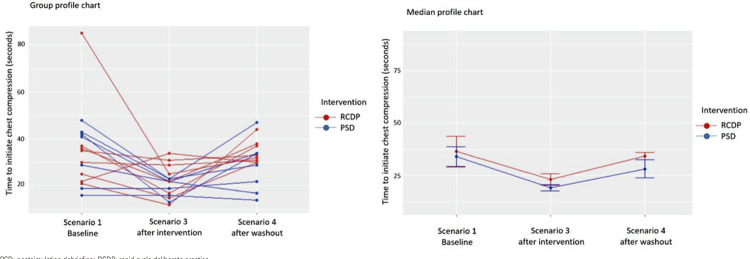
Time to chest compressions

PSD: postsimulation debriefing; RCDP: rapid cycle deliberate practice.

There was a significant decrease in time to initiation of chest compressions from scenario 1 (pre-intervention) to scenario 3 (post-intervention) in both groups. The time to initiation of chest compressions decreased from 36.38 seconds during scenario 1 to 23.1 seconds during scenario 3 in the RCDP Group (p=0.002) and from 31.4 seconds during scenario 1 to 19.1 seconds during scenario 3 in the PSD Group (p=0.027). However, a substantial increase in this same variable was observed after the 5-6-week washout period from scenario 3 to scenario 4. In fact, time to initiation of chest compressions increased from 23.1 seconds during scenario 3 to 34.4 seconds during scenario 4 in the RCDP Group (p=0.003) and from 19.1 seconds during scenario 3 to 28.1 seconds during scenario 4 in the PSD Group (this difference was not statistically significant). Despite significant intergroup differences, no interaction effects or group effects were observed (Figure 3, Table 2).

**Table 2:** Time to chest compressions

	Scenario 1 Baseline	Scenario 3 After the intervention	Scenario 4 After the washout period
Time to initiation of chest compressions (seconds)			
RCDP	36.38 (20.6)	23.1 (7.99)	34.4 (4.93)
PSD	31.38 (13.9)	19.1 (3.98)	28.1 (11.45)

No interaction effects (p=0.885) or group effects (p=0.329) were observed. Variations in the time to chest compressions were observed during the scenarios (p=0.006). This time decreased between scenario 1 and scenario 3 (p=0.024); however, it increased between scenario 3 and scenario 4 (p=0.014). No differences were observed between scenario 1 and scenario 4 (p=0.658).

PSD: postsimulation debriefing; RCDP: rapid cycle deliberate practice.

#### Secondary outcomes

In both groups, there were significant decreases in all times to the performance of tasks between scenario 1 and scenario 3 that were analyzed as secondary outcomes. The mean time to defibrillation, *e.g*., decreased from 160.1 seconds to 92.3 seconds in the RCDP Group (p<0.001), and it decreased from 152.2 seconds to 97.6 seconds in the PSD Group (p=0.008). The time to recognition of CPA, time to checking the rhythm on the monitor, and time to initiation of chest compressions after defibrillation also significantly decreased between scenario 1 and scenario 3 in both groups. However, these times increased after the washout period (scenario 4). The mean time to defibrillation, for example, increased from 92.3 seconds to 147.1 seconds in the RCDP Group (p=0.001), and it increased from 97.6 seconds to 105 seconds in the PSD Group (this difference was not statistically significant). Despite the intergroup differences, no interaction effects or group effects were observed. Regarding the quality of chest compression variables (chest compression rate, chest compression depth, chest recoil) and the chest compression fraction, statistically significant differences were not observed between groups immediately after the intervention and after the washout period.

## DISCUSSION

After training, the teams in both arms exhibited improved times to the performance of critical tasks. This study demonstrated a significant decrease in the time to initiation of chest compressions immediately after RCDP and PSD training among the teams of pediatric residents. Times to the performance of other critical tasks, such as time to defibrillation, also decreased from the pre-intervention to post-intervention scenarios. However, no interaction effects or group effects were observed, suggesting that both simulation methods resulted in similar improvement in pediatric CPR performance immediately after training.

In contrast, the results observed after the washout period implied deterioration in the simulated pediatric CPR performance, as demonstrated by the significant increase in the times to the performance of critical interventions. Nonetheless, no significant differences were observed between groups (no interaction effects or group effects were noted), possibly indicating that both arms of the study experienced similar progression in simulated pediatric CPR performance retention after 5 weeks.

Rapid cycle deliberate practice has been widely used for pediatric CPR training.^(24,25)^ Previous studies have reported the benefits of RCDP training for improving the simulated pediatric CPR performance among residents. Hunt et al. demonstrated a significant improvement in the time to initiation of chest compressions and the time to defibrillation during simulated CPA among pediatric residents after RCDP training.^(13)^ Swinger et al. built upon these findings and reported a significant improvement in pediatric advanced life support performance by pediatric residents after RCDP training as well as good retention of pediatric advanced life support performance 3 months after a single RCDP training session; however, no control groups were evaluated.^(26)^

Compared with PSD, RCDP resulted in equal or superior immediate resuscitation outcomes after training. However, the improvement in simulated CPR performance was not retained over time. During neonatal resuscitation simulation training, RCDP was demonstrated to be superior to PSD in terms of improved abilities and decreased times to the performance of critical interventions among interns.Nontheless,, RCDP was not superior to PSD in terms of improving confidence and retention of improvement after a 4-month period.^(16)^

As for pediatric resuscitation, Lemke et al. reported greater improvement in team performance regarding human factors for RCDP- trained teams when compared to PSD.^(18)^ Furthermore, Cory et al. showed that RCDP was more effective than PSD in training pediatric residents to apply sepsis algorithms immediately after the training period. However, the results regarding retention after a 3-month period were unclear, and deterioration in performance was observed in both arms during follow-up.^(17)^

Won et al. analyzed the times to the performance of critical interventions for pediatric resuscitation, and showed that team leaders trained with RCDP were more likely to perform defibrillation within 3 minutes of cardiac arrest than those who were trained with PSD. In this same study, chest compressions were initiated sooner by the RCDP Group than by the PSD Group; however, the difference was not statistically significant. The results of the skill retention assessment after a 3-month interval were found to be inconclusive.^(20)^

Our findings are consistent with those of previous studies demonstrating significant improvement in pediatric simulated CPR performance immediately after RCDP training and significant performance decay over time. However, no differences were observed between the two study arms in terms of immediate post-intervention results and performance retention after the 5-week washout period. These results suggest both methods can be used to effectively train pediatric residents to perform simulated pediatric CPR, and that the time intervals between training sessions should be reevaluated to optimize retention. Our finding of performance decay after 5 weeks is consistent with that of a study by Anderson et al. which showed that monthly training was more effective in enhancing CPR performance compared to training every 3, 6, or 12 months.^(22)^ Nonetheless, we did not demonstrate the superiority of either method in terms of simulated pediatric CPR performance improvement immediately after training and retention after 5 weeks. Although previous data suggested deterioration in pediatric CPR performance over a 3-month period and the superiority of RCDP over PSD in terms of specific outcomes of simulated pediatric CPR performance, the study designs, study populations, and outcome measures varied between studies. Additionally, feedback and debriefing may have varied across instructors, simulation sessions, and teams.

This study has some limitations. First, it was conducted at a single institution; therefore, generalizability of the results could be compromised. Second, although the residents were part of the same team, they had multiple roles during the simulation sessions. Whether cross-training could be associated with simulated pediatric CPR performance progression over this study is unclear. All participants were pediatric residents; therefore, they performed tasks traditionally assigned to other healthcare providers, such as nurses or respiratory therapists. This may have compromised the realism of the simulation and its reproducibility. Third, some of the participants could not be evaluated during round 2, and one team from the PSD arm was lost to follow-up after round 1. This slightly changed the size of some teams and the proportion of teams in each arm. These variations may have affected the results regarding simulated pediatric CPR retention. Finally, regarding the simulation environment, to accommodate the residents’ schedules, simulation sessions were performed in different settings (simulation center and in situ in the emergency room of the pediatric inpatient unit) with similar available equipment and the same mannequin. This may have impacted the simulated CPR performance; however, this should have had similar effects on each arm because all teams participated in simulations in both settings (either during round 1 or during round 2).

Future studies should compare RCDP and PSD to assess retention of simulated pediatric CPR performance improvement after longer time intervals following training, such as 3, 6, and 12 months. In addition, future research is needed so as to compare these two modalities regarding translation into clinical practice and patient outcomes.

## CONCLUSION

Our study demonstrated that simulated pediatric cardiopulmonary resuscitation performance improved after training with rapid cycle deliberate practice and postsimulation debriefing; however, simulated pediatric cardiopulmonary resuscitation performance in both groups deteriorated after a 5-week interval. No statistically significant differences were observed between these methods immediately after the intervention or during the postwashout period.
